# A modified version of the mental health literacy scale (MHLS) in Iranian people

**DOI:** 10.1186/s12888-021-03050-3

**Published:** 2021-01-23

**Authors:** Mahbobeh Nejatian, Hadi Tehrani, Vahideh Momeniyan, Alireza Jafari

**Affiliations:** 1 Social Determinants of Health Research Center, Gonabad University of Medical Sciences, Gonabad, Iran; 2 Department of Health Education and Health Promotion, Social Determinants of Health Research Center, Mashhad University of Medical Sciences, Mashhad, Iran; 3 Department of Psychology, Ferdowsi University of Mashhad, Mashhad, Iran; 4 Department of Health Education and Health Promotion, Student Research Committee, Mashhad University of Medical Sciences, Mashhad, Iran

**Keywords:** Mental health literacy, Validity, Reliability, Psychometric, Confirmatory factor analysis, Measures, Public population, Persian, MHLS

## Abstract

****Background**:**

The risk rate for the lifetime prevalence of any mental disorder is calculated as 50%, and the prevalence of mental disorders has an increasing trend. So, this study aimed to evaluate the Mental Health Literacy Scale (MHLS) among Iranian people.

****Methods**:**

This cross-sectional study was conducted with a multi-stage sampling method with 1273 people in the general population. After searching and reviewing various sources, the research team decided to use the questionnaire of MHLS with 35 items and six attributes that were measured and developed by O’Connor et al. The face, content, and construct validity (Confirmatory factor analysis) were used for validation of MHLS. McDonald’s omega coefficient and Cronbach’s alpha coefficient were used to calculate the reliability of MHLS. Confirmatory factor analysis was performed using AMOS software Version 24.

**Results:**

In the CFA test, the six items were deleted. The final modified version of the MHLS included a total of 29 items with six attributes consisted of (a) knowledge of where to seek information (4 items), (b) ability to recognize disorders (8 items), (c) knowledge of self-treatment (2 items), (d) knowledge of risk factors and causes (2 items), (e) attitudes that promote recognition or appropriate help-seeking behavior (10 items), and (f) knowledge of professional help available (3 items). Based on the results of reliability, McDonald’s omega coefficient and Cronbach’s alpha coefficient for all attributes of MHLS were 0.797 and 0.789, respectively.

**Conclusion:**

Due to the lack of appropriate instruments for measuring mental health literacy in the Iranian population, the modified version of MHLS with 29 items and six attributes can be considered as a valid and reliable instrument for this purpose.

## Background

World Health Organization (WHO) reported that 8% (586 million) of the global population suffer from mental disorders [1]. The results of a study performed in China showed that the prevalence rate of severe mental illness in 1-month and lifetime is 9.35 and 10.10%, respectively [2]. Moreover, based on the results of a meta-analysis conducted in Iran, the prevalence rate of psychiatric disorders was reported as 31.03% [3].

The frequency of mental disorders among the general population means that many people could directly face a mental health problem in their families, but most of them do not have enough knowledge and skills to assist them [4]. Generally, mental disorders of the individuals can cause shorter lifetime [5]. Mental health literacy (MHL) is considered as a significant predictor of favorable health outcomes [6]. The term of MHL was firstly used in 1997 to describe knowledge and beliefs related to mental disorders that help in diagnosing, managing, and preventing them. The increased general knowledge, as a prerequisite for early diagnosis and intervention in mental disorders, is required on the concept of mental health and its related disorders [4, 7, 8].

MHL refers to “focusing on knowledge and strategies to obtain and maintain a good mental health state, knowledge on mental disorders and related treatments, and strategies to decrease stigma and enhance help-seeking efficacy” [6]. Up to now, the studies on mental health have shown that many people have a poor MHL because they have no idea about psychological problems and have negative attitudes about their treatment or effectiveness of the treatments. However, having a high MHL also has several advantages such as prevention from the disease, early diagnosis of symptoms, and performing the necessary interventions to reduce symptoms of mental disorder [4]. The results show that if people have positive attitudes on help-seeking and perceive need for treatment significantly and independently, the use of psychotherapy can be predicted over time by them [9].

The results of a systematic study in Iran showed that 32% of women had not an adequate level of health literacy. Also, the level of health literacy was low in women with chronic diseases [10]. The results of another systematic study showed that the health literacy status of the Iranian people was inadequate and borderline [11]. The results of a study on Iranian medical sciences students showed that 64.4% of people were unable to recognize the mental disorder, and 36% did not know where to help-seeking about mental disorders [12]. Another study conducted in Iran showed that depression literacy was low, and 48.5% of participants cannot recognize the mental disorder, and 47.15% didn’t intend to seek help ([13]. The results of another study on the general population in Iran showed that mental health literacy status is not sufficient [14].

Given the increasing prevalence of mental disorders and the important role of mental health literacy in reducing these disorders, a suitable tool is needed to measure the level of mental health literacy in the community. However, up to the time of performing this study, no valid and reliable instrument was provided for measuring MHL in Iranian people. Accordingly, all available instruments in Iran could just measure general health literacy and could not specifically measure MHL. After searching and reviewing various sources, the research team decided to use the questionnaire of MHLS with 35 items and six attributes developed by O’Connor et al. [15]. MHL includes six attributes includes of the ability to recognize specific disorders, knowledge of professional help available, knowing how to seek mental health information, knowledge of self-treatments, knowledge of risk factors and causes, and attitudes that promote recognition and appropriate help-seeking [16]. The availability of a valid tool can help people diagnose mental disorders in the early stages, and seek available treatment. Therefore, this study aimed to evaluate the MHLS in the general population of Gonabad, Iran.

## Methods

This cross-sectional study was conducted to evaluate the validity and reliability of the Iranian version of the MHLS with 1273 individuals of the general population in Gonabad, Iran in 2019.

### Sample size and sampling method

The sample size with the 0.95% confidence level, proportion 0.48 the accuracy of 0.03, and Considering 20% of sample loss, was estimated at 1330 subjects [13]. In this study, 1330 questionnaires were distributed among the participants. Finally, 1273 questionnaires were returned, of which 57 questionnaires were excluded from the study due to incomplete information. The final analysis was performed on 1273 participants, and the response rate in this study was 96%.

In this study, the participants were selected by multistage sampling. Initially, the number of community health centers and the population of each one of them were determined. In the next step, these centers were stratified as follows: each center was considered as one category and the sample size was determined due to the population of each category. Finally, the participants were randomly selected from each center. In the present study, the interviewers completed the questionnaire for illiterate participants. The inclusion criteria were the followings: age over 18 years old, having no physical or mental disorder, signing the written informed consent to participate in the study, and being resident of Gonabad city.

### Instruments

Data collection tools included a demographic section and Mental Health Literacy Scale (MHLS).

#### Demographic questionnaire

This questionnaire includes questions on gender, age, occupation, level of education, marital status, etc.

#### MHLS

This questionnaire was the development and evaluation by O’Connor et al. in 2015 [15].

The MHLS is a single-factor measure. This questionnaire has 35 questions and six attributes. While there are items related to the relevant attributes, they are intended to be considered together. Also, these attributes were review and adapted with studies of Jorm [17], Griffiths, et al., [18], and Jorm, et al. [16].

A. **Ability to recognize disorders:** This attribute consists of eight questions that were measured using a 4-point Likert scale (very unlikely, unlikely, likely, very likely). This attribute refers to “the ability to correctly identify features of a disorder, a specific disorder, or category of disorders”.
B. **Knowledge of risk factors and causes:** This attribute was measured with two questions and using a 4-point Likert scale (very unlikely, unlikely, likely, very likely). This attribute refers to “knowledge of environmental, social, familial or biological factors that increase the risk of developing a mental illness”.
C. **Knowledge of self-treatment:** was measured This attribute consists of two questions that were measured using a 4-point Likert scale (very unhelpful, unhelpful, helpful, very helpful). This attribute refers to “knowledge of typical treatments recommended by mental health professionals and activities that an individual can conduct”.
D. **Knowledge of professional help available:** This attribute was measured with three questions and using a 4-point Likert scale (very unlikely, unlikely, likely, very likely). This attribute refers to “knowledge of mental health professionals and the services they provide”.
E. **Knowledge of where to seek information:** This attribute consists of four questions that were measured using a 5- option Likert scale (strongly disagree, disagree, neither agree nor disagree, agree, strongly agree). This attribute refers to “knowledge of where to access information and capacity to do so”.
F. **Attitudes that promote recognition or appropriate help-seeking behavior:** This attribute consists of sixteen questions and were measured using a 5-option Likert scale [(strongly disagree, disagree, neither agree nor disagree, agree, strongly agree) or (definitely willing, probably willing, neither willing nor unwilling, probably unwilling, definitely unwilling). This attribute refers to “attitudes that impact on the recognition of disorders and willingness to engage in help-seeking behavior”.

In this questionnaire, the lowest score is 35, the highest score is 160, and higher scores indicate a better MHL status. The validity and reliability of this questionnaire were evaluated in the O’Connor study. The internal consistency of this scale was measured by Cronbach’s alpha (Cronbach’s alpha= 0.873) [15].

### Translation and cultural adaptation

In this research, we used the forward-backward method to do the translation and cultural adaptation [19]. At first, the original English version of the questionnaire was translated into the Persian language by two experts separately. Afterward, the two translated versions were reviewed and a single Persian version of the questionnaire was then prepared. Subsequently, one expert in English language who was not familiar with the specialized English text of psychology back-translated the text into English. Thereafter, the English text of the backward-translation version was adapted with the original English version of the questionnaire. Finally, the English translation was re-translated into Persian language by three psychology specialists who were expert in English language. Moreover, the validity and reliability of the questionnaire were evaluated.

### Validation

Given the reason that the standard questionnaire has been used and translated in this study, quantitative content and face validities were not required to be measured [20]. Furthermore, in this study, the validity of the questionnaire was assessed by qualitative face and content validities.

### Validity (qualitative of face and content validity)

To assess the face validity, face-to-face interviews were conducted with some of the participants (*n*=8) to find out any difficulty in understanding the words and phrases, the appropriateness and relevance of the items, the likelihood of ambiguity and misunderstandings, or any failure in conceptualization. In case of any problem, the participants’ comments were applied to the questionnaire.

To assess the content validity, the questionnaire was provided to 10 specialists (panel of experts in the fields of psychology, health literacy, and health education and promotion) for the purpose of evaluating grammar, use of appropriate words, the importance of items, the correct placement of items, and the time for completing the designed instrument in the present study. After collecting the expert evaluations’ results, necessary changes were made in consultation with the members of the research team.

### Confirmatory factor analysis (CFA)

CFA was used to evaluate the construct validity. Before CFA, the obtained data were analyzed using Mahalanobis statistics for the outliers. Subsequently, the normality of the data was evaluated using skewness and kurtosis. CFA was then performed using AMOS version 24 software. Subsequently, the items with weak internal consistency were removed from the questionnaire to obtain an acceptable model. Based on the results, to find an acceptable final model, those questions with a factor loading less than 0.3 were deleted [21].

The assessment of the model was conducted with using the following fit indices: Chi-square ratio to the degree of freedom (× 2/df); root means square residual (RMR); root means the square error of approximation (RMSEA); goodness of fit index (GFI); adjusted goodness of fit index (AGFI); parsimonious normed fit index (PNFI); parsimony comparative fit index (PCFI); incremental fit index (IFI); parsimony goodness-of-fit index (PGFI); comparative fit index (CFI); and parsimonious normed fit index (PNFI) [22–24]. The model was acceptable if the (× 2/df) < 5, RMSEA and RMR ≤ 0.08, PCFI, PNFI and PGFI> 0.5, AGFI > 0.8, and other indices of IFI, GFI, CFI > 0.9 [22–25].

### Reliability assessment

**Fig. 1:**
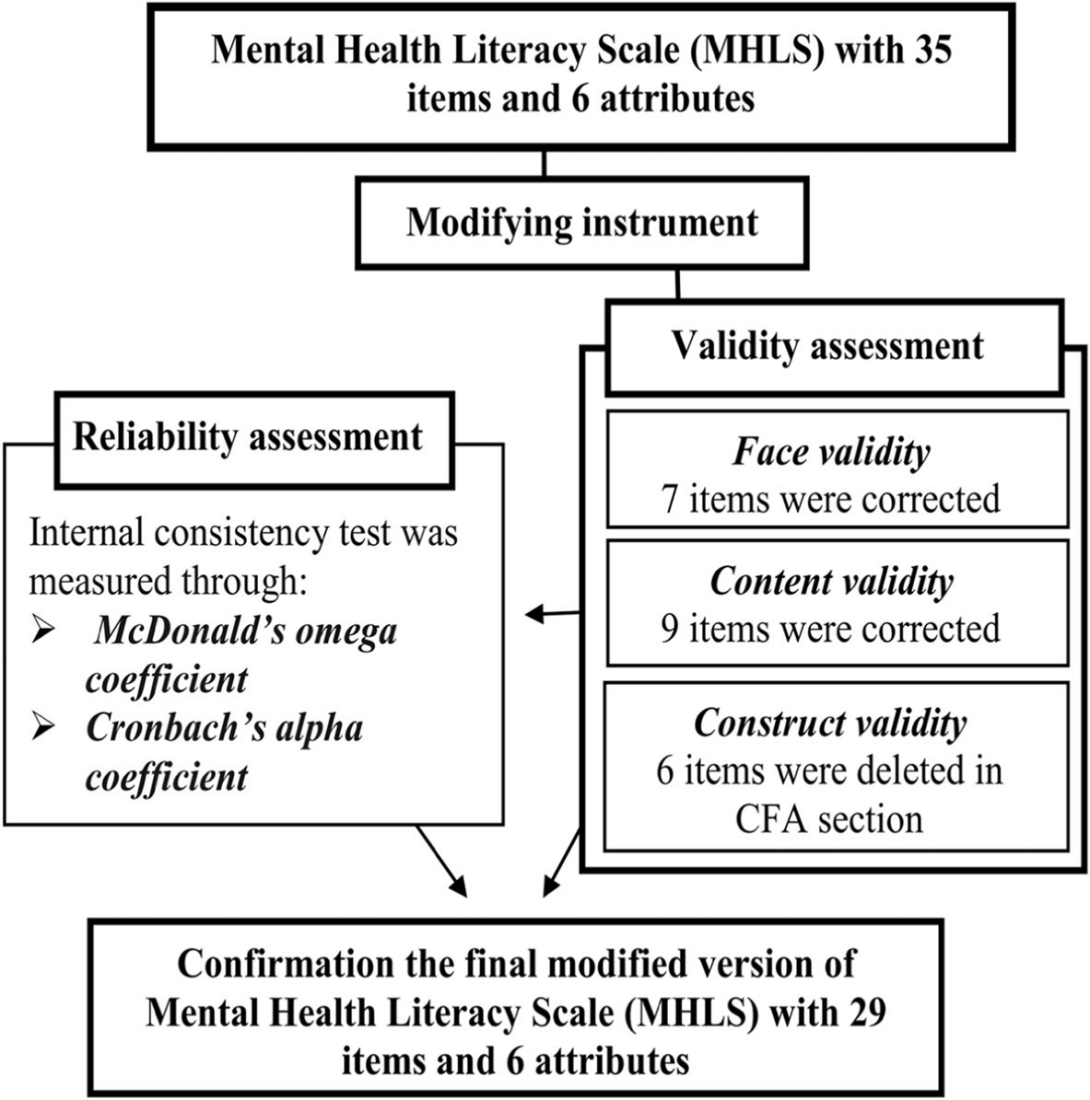
A summary of the modifying of MHLS

McDonald’s omega coefficient and Cronbach’s alpha coefficient were used to assess the internal consistency of the questionnaire and each of the attributes separately. The JASP (Version 0.11.1) software and SPSS_v22_ were used to calculate the amount of McDonald’s omega coefficient and Cronbach’s alpha coefficient, respectively. McDonald’s omega coefficient provides a more accurate approximation than Cronbach’s alpha coefficient [26]. Based on the results, when developing a new measure, the value of the reliability coefficient above 0.70 is routinely considered acceptable [27]. Based on the results of the Wallston study, Cronbach’s alpha coefficient of 0.6 was considered as the minimum acceptance criterion for the internal reliability of the questionnaire [28]. The lower values of McDonald’s omega coefficient and Cronbach’s alpha coefficient be related to their low number of items is some attributes [27]. A summary of the modifying of MHLS is presented in Fig. 1.

## Results

The mean (standard deviation) age of the participants in this study was 31.17 (10.13), and 58% (*n*=732) of participants were female, and 68.8% (*n*=864) were married. Most of them had an associate/bachelor’s degree (57.9%) and a high school diploma (29.9%). In this study, 79.5% (*n*=936) of the participants were residents in the city, and most of them were self-employed (38.4%) (Table 1). The mean (SD) of the total MHLS was 97.99 (11.47).

**Table 1:** Frequency distribution of demographic characteristics (*n* = 1273)

Variables		N	%
**Gender**	Male	531	42
	Female	732	58
**Marital status**	Marriage	864	68.8
	Single	391	31.2
**Education level**	Elementary	38	3.2
	Diploma	366	29.9
	Associate or Bachelor’s Degree	708	57.9
	Master’s degree or High degree	109	9
**Residence**	Urban	936	79.5
	Rural	242	20.5
**Occupation**	Housewife	211	17.4
	Employed	363	30
	Self-employed	464	38.4
	labor	101	8.3
	Unemployed	71	5.9

### Validity (qualitative of face and content validity)

No question was omitted during the translation and cultural adaptation processes because the subject’s statements in the original questionnaire were similar to the culture of the Iranian population. During the processes of face and content validities’ assessment, the questionnaire was given to 10 specialists (panel of experts in the fields of psychology, health literacy, and health education and promotion). As a result, seven and nine items that had difficulties in understanding of the words and grammatical problem, were corrected in face and content validities, respectively (Fig. 1).

### Confirmatory factor analysis (CFA)

**Fig. 2:**
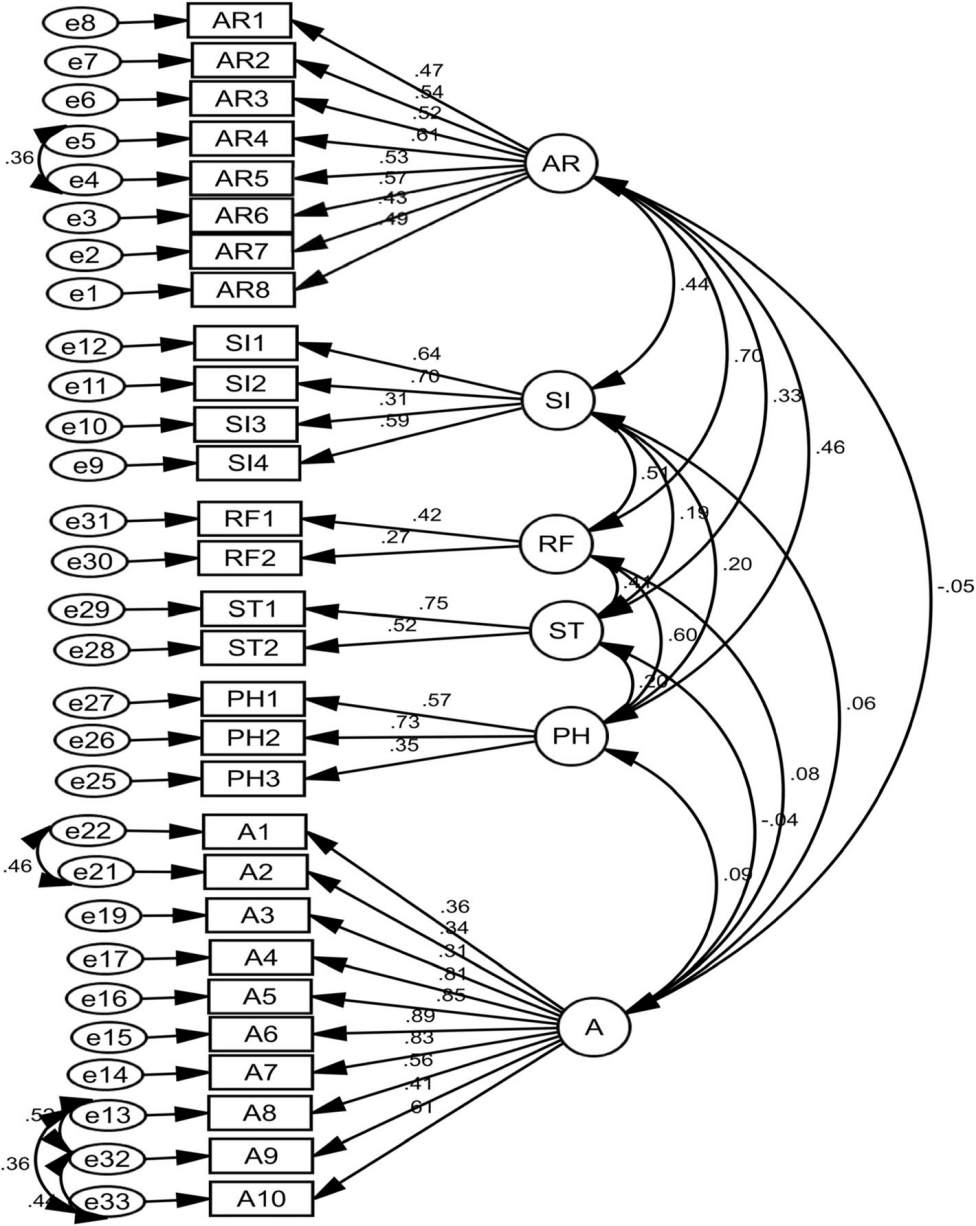
Standardized parameter estimates for the factor structure of the MHLS

The results of the CFA analysis showed that the CR value of each question was above 1.96 and the significance level of all questions was less than 0. 001. The goodness of fit for these six attributes model was acceptable: X2/df=4.672, RMR=0.047, RMSEA=0.054, PCFI=0.772, PGFI=0.749, PNFI=0.748, AGFI=0.893, GFI=0.913, CFI=0.901, and IFI=0.901 (Table 2). In the CFA stage, to obtain an acceptable final model, six questions with a factor loading less than 0.3 were deleted (Table 3). The factor loading value of each question is visible in Table 3 and Fig. 2.

**Table 2:** The model fit indicators of MHLS

Goodness of fit indices	Confirmatory factor analysis	Acceptable value
**X**^**2**^	1667.778	–
**df**	357	–
**X**^**2**^**/df**	4.672	< 0.5
***p*-value**	0.001	*p* > 0.05
**CFI**	0.901	> 0.9
**IFI**	0.901	> 0.9
**GFI**	0.913	> 0.9
**AGFI**	0.893	> 0.8
**RMSEA**	0.054	< 0.08
**RMR**	0.047	< 0.08
**PNFI**	0.748	> 0.5
**PCFI**	0.772	> 0.5
**PGFI**	0.749	> 0.5

**Table 3:** Factor loadings of the MHLS in the CFA stage

Attributes	Items	Factor loadings
**Ability to recognize disorders (AR1-AR 8)**	1. If someone gets very anxious and nervous in one or more situations in front of other people, for example at a party or when he has to do something (such as giving a speech in a meeting), in which they were afraid of others or feel ashamed, how much do you think that person has Social Phobia.	0.468
	2. If a person is very worried about several events and activities, where this level of concern was not warranted, cannot control this worry, and had physical symptoms (such as muscle cramps and feeling tired), how much do you think this person has a Generalized Anxiety Disorder.	0.537
	3. If a person had a low mood for two or more weeks, not interested in normal and daily activities, and feels changes in his appetite and sleep, how much do you think this person has **Major Depressive Disorder.**	0.519
	4. To what extent do you think **Personality Disorders** can be classified as a mental illness.	0.615
	5. How likely do you think Dysthymia is a disorder.	0.531
	6. To what extent do you think being anxious in situations and places where escaping is difficult or embarrassing can be diagnosed as a “Agoraphobia”.	0.575
	7. How likely do you think the diagnosis of Bipolar Disorder includes experiencing a period of happy mood and periods of sad mood (depression) by someone.	0.433
	8. To what extent do you think physical and psychological tolerance for drugs (need for more substances to maintain its effect on the body) may be identified as drug dependence and addiction.	0.494
**Knowledge of risk factors and causes (RF1,RF2)**	1. In general, to what extent do you think women in Iran may be more **experience** any mental illness than men.	0.422
	2. In general, to what extent do you think men in Iran may be more **experience** anxiety disorders more than women.	0.270
**Knowledge of self-treatment (ST1,ST2)**	1. If someone has difficulty managing their emotions (for example, becoming very anxious or depressed), how much do you think improving their sleep quality can be beneficial to them?	0.754
	2. If someone has difficulty managing their emotions (for example, becoming very anxious or depressed), how much do you think avoiding all activities or situations that made them feel anxious can be beneficial to them.	0.521
**Knowledge of the professional help available (PH1-PH3)**	1. In your opinion, it is likely that that **Cognitive Behavior Therapy** is a therapy based on challenging negative thoughts and increasing helpful behaviors.	0.575
	2. Mental health professionals are bound by confidentiality; however, there are certain conditions under which this does not apply. To what extent do you think it is likely that the following is a condition that would allow a mental health professional to break confidentiality: *-If you are at risk of harming yourself or others, how likely do you think a Mental health professional will reveal your secrets to others?*	0.731
	3. Mental health professionals are bound by confidentiality; however, there are certain conditions under which this does not apply. To what extent do you think it is likely that the following is a condition that would allow a mental health professional to break confidentiality: *-If your problem does not a serious threat to your life and Mental health professionals want to get assist others to better support you, how likely do you think a Mental health professional will tell your secrets to others?*	0.354
**Knowledge of where to seek information (SI1-SI4)**	1. I’m sure I know where to look for information about mental disorders.	0.639
	2. I’m sure I can use computers and telephones to seek information about mental disorders.	0.699
	3. I’m sure attending face to face meeting to seek information about mental disorders (e.g., seeing the general practitioner, psychologist).	0.307
	4. I am confident that I have access to resources such as the Internet, general practitioner, friends, etc. to seek information about mental disorders.	0.592
**Attitudes that promote the recognition or appropriate help-seeking behavior(A1-A10)**	1. People with mental disorders are dangerous.	0.355
	2. It is best to avoid people with a mental disorder so that you don’t develop this problem	0.344
	3. If I have a mental disorder, I do not like to tell anyone.	0.310
	4. How much do you want to move next door to someone with a mental illness?	0.807
	5. How much do you want to spend the night with someone who has a mental disorder?	0.850
	6. How much do you want to be friends with someone who has a mental disorder?	0.887
	7. How much do you want to start working with someone who has a mental disorder?	0.826
	8. How much do you want to have someone with a mental illness marry into your family?	0.555
	9. How much do you want to vote for a politician who suffering from a mental illness?	0.410
	10. How much do you want to employ someone with a mental illness?	0.613
	➢ People with a mental disorder could stop their behaviors if they wanted.	Deleted
	➢ A mental disorder is a sign of personality weakness.	Deleted
	➢ A mental disorder is not a real medical disease.	Deleted
	➢ If I have a mental d**isorder**, I do not like to seek help from a mental health professional (e.g., psychologist/psychiatrist).	Deleted
	➢ I believe that the treatment of mental d**isorders** by a mental health professional (e.g., psychologist/psychiatrist) is ineffectiveness.	Deleted
	➢ Refer to a psychologist means that you do not have enough power to manage and solve your problem.	Deleted

The final version of the MHLS included a total of 29 items, which consisted knowledge of where to seek information (4 items), knowledge of self-treatment (2 items), ability to recognize disorders (8 items), attitudes that promote recognition or appropriate help-seeking behavior (10 items), knowledge of risk factors and causes (2 items), and knowledge of professional help available (3 items) (Table 4). Details attributes of MHLS are available in Table 4.

**Table 4:** Descriptive statistics of the MHLS and its attributes

Attributes	Item	Mean	SD	Range	Cronbach’s alpha	McDonald’s omega
**Ability to recognise disorders (AR)**	8	23.81	3.52	8–32	0.700	0.734
**Knowledge of where to seek information (SI)**	4	13.65	2.71	4–20	0.630	0.652
**Knowledge of risk factors (RF)**	2	5.39	1.07	2–8	0.600	0.601
**Knowledge of self-treatment (ST)**	2	5.15	0.84	2–8	0.600	0.602
**Knowledge of professional help available (PH)**	3	8.43	1.15	3–12	0.640	0.643
**Attitudes that promote recognition or appropriate help seeking behaviour(A)**	10	36.58	5.30	10–50	0.800	0.874
**The final modified version of MHLS (All attributes)**	29	93.03	8.16	29–130	0.789	0.797

### Reliability

McDonald’s omega coefficient for all questions of MHLS, MHLS attributes includes the ability to recognize disorders, knowledge of where to seek information, knowledge of risk factors, knowledge of self-treatment, knowledge of professional help available, attitudes that promote recognition or appropriate help-seeking behavior, were 0.797, 0.734, 0.652, 0.601, 0.602, 0.643, and 0.874 respectively (Table 4). Cronbach’s alpha coefficient for all questions of MHLS, MHLS attributes includes the ability to recognize disorders, knowledge of where to seek information, knowledge of risk factors, knowledge of self-treatment, knowledge of professional help available, attitudes that promote recognition or appropriate help-seeking behavior, were 0.789, 0.700, 0.630, 0.600, 0.600, 0.640, and 0.800, respectively (Table 4).

## Discussion

This study aimed to evaluate the Mental Health Literacy Scale (MHLS) among Iranian people. It is noteworthy that there is no specified instrument for evaluating MHL in Iran up to now, and besides, no study was conducted on psychometrically the MHLS. One of the features of the instrument proposed in this study is measuring different aspects of MHL with a short time and with self-administration. In the present study, this instrument was completed by most of the participants with no problem in a short time. Therefore, this instrument seems to be useful for measuring the MHL of different age groups in society. Accordingly, this tool can be used to measure MHL, to identify individuals with a low level of literacy in any attribute and design, and to implement intervention programs for them.

In the present study, this 35 items questionnaire was evaluated and modified. After evaluation of the questionnaire, six questions were omitted, and the modified version of MHLS with 29 items and six attributes was approved. In the present study for assessing the reliability of the instrument, McDonald’s omega coefficient, and Cronbach’s alpha coefficient were used and calculated 0.797 and 0.789, respectively. McDonald’s omega coefficient similar to Cronbach’s Alpha, but the omega coefficient provides a more accurate approximation of a scale’s reliability, and that the omega coefficient is almost always higher than Cronbach’s alpha coefficient [26]. Based on the results of many previous study, McDonald’s omega coefficient is a more sensible index of internal consistency compared to Cronbach’s alpha and other alternatives [26, 29, 30]. Based on these results, when developing a new measure, the value of the reliability coefficient above 0.70 is routinely considered as acceptable [27].

In a study by Noroozi, the Cronbach’s alpha for total attributes of MHLS was 0.74 [31]. Moreover, in a study conducted by O’Connor, the MHLS was designed based on some other questionnaires in this field. In this regard, the 55-item questionnaire was evaluated, in which the MHLS with 35-item and six attributes were finally confirmed after psychometric evaluation of the questionnaire. Accordingly, Cronbach’s alpha of 0.879 and test-retest reliability of 0.797 were reported [15].

In the CFA stage of the present study, the six items were deleted. The final version of the MHLS was approved with 29 items and included six attributes of ability to recognize disorders (8 items), knowledge of where to seek information (4 items), knowledge of risk factors and causes (2 items), knowledge of self-treatment (2 items), knowledge of professional help available (3 items), and attitudes that promote recognition or appropriate help-seeking behavior (10 items). Jung conducted a study aimed to developing and assessing the reliability of an instrument used for evaluating MHL. Correspondingly, the results of the exploratory factor analysis discovered three factors for the 26-item questionnaire. Moreover, the results of CFA showed that the proposed model has a good fit in the stage of CFA. Also, Cronbach’s alpha amounts were reported as 0.76, 0.77, and 0.84 for the first factor (knowledge-oriented MHL), the second factor (Beliefs-oriented MHL), and the third factor (resource-oriented MHL), respectively [32]. The results of a systematic review that examined the tools available in the field of the evaluation of MHL, showed that the MHLS used in the present study is an acceptable tool for evaluating MHL in individuals [33].

The first attribute of this instrument was “the ability to recognize disorders”. This attribute was confirmed by eight items, Omega 0.734, alpha 0.700, and factor loading 0.433 to 0.615. Finding the appropriate tools for early diagnosis of various types of mental disorders is important. The results of a study conducted by Jorm in Australia showed that people who had a better ability in recognizing depression and schizophrenia also were more likely to receive a wide range of interventions including assistance from mental health professionals, psychotherapy, medications, and psychiatric admissions [34].

The second attribute of this instrument was “knowledge of where to seek information”. This attribute was confirmed by four items, Omega 0.652, alpha 0.630, and factor loading 0.639 to 0.699. The results of a study performed in China have also indicated that people have great intentions to seek mental health services. However, they have low levels of knowledge on help sources and no knowledge about where to seek potential help sources [35]. Also, the results of a recent systematic review study have shown the improved knowledge on mental disorders/mental health, where to seek help and treatment, the improved the mental health outcomes, and the increased use of mental health services by people [36].

The third attribute of this instrument was “knowledge of risk factors”. This attribute was confirmed by two items, Omega 0.601, alpha 0.600, and factor loading 0.270 to 0.422. Undoubtedly, one of the less well-regarded aspects of MHL is prevention. We have more knowledge about the risk factors for other diseases compared to the risk factors of mental disorders, and people should also have access to appropriate tools to identify modifiable risk factors for mental disorders [37]. It was indicated that people who have access to the appropriate tools to diagnose risk factors can better manage their preventive behaviors [38].

The fourth attribute of this instrument was “knowledge of self-treatment”. This attribute was confirmed by two items, Omega 0.602, alpha 0.600, and factor loading 0.516 to 0.752. The ability to diagnose a mental disorder is useful, but the individual must also have knowledge on the available evidence-based treatments [37]. The results of the Thompson’s study in Australia showed that the most important reason for psychiatric patients to delay treatment is the lack of knowledge on available treatments [39].

The fifth attribute of this instrument was “knowledge of professional help available”. This attribute was confirmed by three items, Omega 0.643, alpha 0.640, and factor loading 0.354 to 0.731. Another important attribute of MHL is knowledge about professional help available in the community for the treatment of mental disorders [39]. Based on the results, most of the people with a mental disorder receive no treatment from health care service because they do not know how to access an available treatment [40].

The sixth attribute of this instrument was “attitudes that promote recognition or appropriate help-seeking behavior”. This attribute was confirmed by ten items, Omega 0.874, alpha 0.800, and factor loading 0.355 to 0.887. Knowing the status of individuals’ attitudes that promote recognition or appropriate help-seeking behavior, is essential to prevent some manners like labeling. Therefore, an appropriate tool that can examine people’s attitudes can be effective on the processes of prevention and treatment of mental disorders. Findings of a study by Reynders showed that people who have more positive attitudes toward help-seeking and experiencing less self-stigma, have fewer psychological problems, which prevent these problems [41].

### Strengths and limitations

One of the limitations of this study was the shorter final modified version of the questionnaire compared to the original version of the questionnaire, which consequently changed the scoring. Since this questionnaire had no level and mental health literacy status was reported as mean (SD), and also given the fact that obtaining a higher score indicates a better mental health literacy status, so it can be justified. One of the strengths of this study was that it was conducted in the general population with different age groups and social classes. The large sample size was another power of this study. Therefore, given the confirmation of the validity of the MHLS in this study and the applicability of this questionnaire to assess the level of MHL in different groups of society, it is recommended to use this questionnaire to assess the MHL of different target groups for educational, clinical, and research purposes. Also, due to the reason that this questionnaire is new, it is recommended to evaluate its psychometric in some other studies with various target populations.

## Conclusions

Based on the results of this study, due to a lack of appropriate tools for evaluating MHL in the Iranian population, the modified version of MHLS with 29 items and six attributes is a suitable instrument for assessing MHL in individuals. Due to the shortness and ease of use, this instrument can be used to measure the level of MHL in different groups of society and to identify people with low MHL. Identifying people with insufficient MHL levels enables mental health services to design and implement appropriate mental health intervention programs and prevent the prevalence of mental disorders in the community.

## Data Availability

The data sets used and/or analyzed during the current study were available from the corresponding author on reasonable request.

## Abbreviations

MHLS: Mental Health Literacy Scale
MHL: Mental Health Literacy
CFA: Confirmatory factor analysis
YLDs: Years lived with a disability
DALYs: Disability-adjusted life years
AR: Ability to recognize disorders
SI: Knowledge of how to seek information
RF: Knowledge of risk factors
ST: Knowledge of self-treatment
PH: Knowledge of professional help available
A: Attitudes that promote recognition or appropriate help- seeking behavior
